# Non-criteria antiphospholipid antibodies and pregnancy outcomes in recurrent pregnancy loss patients

**DOI:** 10.3389/fmed.2026.1961459

**Published:** 2026-10-21

**Authors:** Xiaoling Zhou, Erdan Luo, Xiaohui Cai, Wei Chen, Li Xiao, Congrong Zhou, Tao Wang, Min Xiong, Fan Lai, Yi Deng

**Affiliations:** 1 Department of Pharmacy, Chengdu Women's and Children's Central Hospital, School of Medicine, University of Electronic Science and Technology of China, Chengdu, China; 2 Department of Good Clinical Practice, Chengdu Women's and Children's Central Hospital, School of Medicine, University of Electronic Science and Technology of China, Chengdu, China; 3 Department of Obstetrics and Gynecology, The Affiliated Hospital of Southwest Medical University, Luzhou, China; 4 Department of Traditional Chinese Medicine, Chengdu Women’s and Children’s Central Hospital, School of Medicine, University of Electronic Science and Technology of China, Chengdu, China; 5 Department of Medical Affairs, Chengdu Women’s and Children’s Central Hospital, School of Medicine, University of Electronic Science and Technology of China, Chengdu, China; 6 Department of Obstetrics, Chengdu Women's and Children's Central Hospital, School of Medicine, University of Electronic Science and Technology of China, Chengdu, China

**Keywords:** anti-phosphatidylethanolamine, multiple non-criteria antiphospholipid antibodies positivity, non-criteria antiphospholipid antibodies, pregnancy outcome, recurrent pregnancy loss

## Abstract

**Objective:**

This study seeks to examine the prevalence of non-criteria antiphospholipid antibodies (NC-aPLs) in patients experiencing recurrent pregnancy loss (RPL) and to assess their potential association with pregnancy outcomes.

**Methods:**

In this study,247 patients who had experienced at least two pregnancy losses and had been screened for 14 NC-aPLs were recruited between February 2021 and March 2024. Serum levels of NC-aPLs were assessed using line immunoassay (LIA), with the exception of anti-phosphatidylserine/prothrombin IgM (aPS/PT), which was quantified via enzyme-linked immunosorbent assay (ELISA). Pregnancy outcomes were subsequently compared between patients testing positive and negative for NC-aPLs, following the exclusion of individuals with positivity for criteria antiphospholipid antibodies (aPLs), chromosomal abnormalities, uterine anomalies, or thyroid dysfunction. Binary logistic regression analysis was employed to identify factors associated with adverse pregnancy outcomes (APO).

**Results:**

The overall positivity rate for NC-aPLs was 38.46% (95 out of 247 participants), with anti-phosphatidylethanolamine (aPE) IgM being the most prevalent subtype, observed in 14.17% of cases. Patients who tested positive for NC-aPLs received more intensive therapeutic interventions, including low molecular weight heparin (LMWH), glucocorticoids, and hydroxychloroquine (HCQ). However, the incidence of APO did not significantly differ between NC-aPLs-positive and NC-aPLs-negative groups (53.68% vs. 46.71%, OR = 1.322, 95% CI 0.791–2.211, *p* = 0.286). Subgroup analyses did not reveal any significant associations between specific NC-aPLs subtypes [aPE IgM, anti-prothrombin (aPT) IgM, anti-annexin A5 (aAnxA5] IgM) and APO, nor between single and multiple NC-aPLs positivity. Binary logistic regression showed that medicinal degree was associated with lower odds of APO (1–2 drugs: aOR=0.177, 95% CI 0.055–0.569, *P* = 0.004; 3–4 drugs: aOR = 0.102, 95% CI 0.037–0.283, *P* < 0.001), while neither single positivity (aOR = 1.553, 95% CI 0.799–3.017, *P* = 0.194) nor multiple positivity for NC-aPLs (aOR = 1.942, 95% CI 0.798–4.726, *P* = 0.144) was significantly associated with APO.

**Conclusion:**

NC-aPLs are detectable in a substantial proportion of patients with RPL. However, this study did not identify any association between NC-aPLs and APO. Patients who tested positive for NC-aPLs received significantly more intensive pharmacological treatment, which was associated with lower odds of APO. This confounding factor may obscure the potential effects of NC-aPLs. Additionally, the limited sample size and the use of a single-time LIA for NC-aPLs detection may introduce bias into the current findings. Further research involving large-scale cohorts and repeated standardized antibody testing is necessary to elucidate the clinical relevance of NC-aPLs and APO.

## 1 Introduction

Recurrent pregnancy loss (RPL) is characterized by the failure of two or more clinically recognized pregnancies before reaching 20–24 weeks of gestation (1). This condition affects approximately 1%–5% of couples attempting to conceive and represents a significant reproductive health issue (2), impacting both the physical and psychological well-being of prospective parents (3). The etiology of RPL can include chromosomal abnormalities, antiphospholipid antibody syndrome (APS), uterine anomalies, and abnormal thyroid function (4).

APS is a notable cause of RPL, with its diagnosis necessitating both clinical criteria and persistent laboratory evidence of conventional antiphospholipid antibodies (aPLs), specifically lupus anticoagulant (LA), anticardiolipin antibodies (aCL), and anti-β2-glycoprotein I antibodies (anti-β2GPI) (5). Nevertheless, a significant proportion of patients with RPL testing negative for conventional aPLs, they still exhibit clinical features characteristic of APS (6). This observation has led to increased research into the potential role of non-criteria aPLs (NC-aPLs) in RPL (7).

Recent evidence suggests that various NC-aPLs, including antibodies against phosphatidylethanolamine (aPE), anti-phosphatidylserine (aPS), anti-prothrombin (aPT), anti-phosphatidylcholine (aPC), anti-phosphatidylserine/prothrombin IgM (aPS/PT), anti-phosphatidic acid (aPA), anti-phosphatidylinositol (aPI) and anti-annexin A5 (aAnxA5), may contribute to the pathogenesis of RPL (8, 9). However, the clinical relevance of these antibodies remains a subject of debate. While some studies have identified positive correlations between specific NC-aPLs and pregnancy loss (10), others have failed to establish such findings (11).

In light of these inconsistencies, the current study was conducted with a rigorously defined cohort of RPL patients to: (1) ascertain the prevalence of 14 NC-aPLs, specifically aPS/PT IgG/IgM, aPE IgG/IgM, aPT IgG/IgM, aAnxA5 IgG/IgM, aPS IgG/IgM, aPA IgG/IgM, and aPI IgG/IgM, after thoroughly excluding other known causes of RPL, and (2) evaluate the association of these antibodies with subsequent adverse pregnancy outcomes(APO), including pregnancy loss, stillbirth, preterm delivery, preeclampsia, fetal growth restriction (FGR), and intrauterine distress.

## 2 Materials and methods

### 2.1 Study patients

This was a single-center, retrospective cohort study. Female patients with a history of two or more pregnancy losses were recruited at the Chengdu Women's and Children's Central Hospital between February 2021 and March 2024. A total of 331 patients were screened for the presence of 14 NC-aPLs and had at least one subsequent pregnancy outcome. Based on the following inclusion criteria, 247 patients were subsequently included in the study (see Figure 1) (1) a history of at least two pregnancy losses and (2) testing for aCL IgG/IgM/IgA, aβ2GPI IgG/IgM/IgA, LA, aPE IgG/IgM, aPT IgG/IgM, aPS/PT IgG/IgM, aAnxA5 IgG/IgM, aPS IgG/IgM, aPI IgG/IgM,aPA IgG/IgM. The exclusion criteria were as follows:(1)patients with positivity for criteria antiphospholipid antibodies (aCL, LA, or aβ2GPI),(2)patients with chromosomal abnormalities in either partner,(3)patients with uterine anomalies such as uterine mediastinum, intrauterine adhesions, or bicornuate uterus,(4) those with abnormal thyroid function such as hyperthyroidism, or hypothyroidism, and (5) those lost to follow-up. The study protocol received approval from the Chengdu Women's and Children's Central Hospital Ethics Committee.

**Figure 1:**
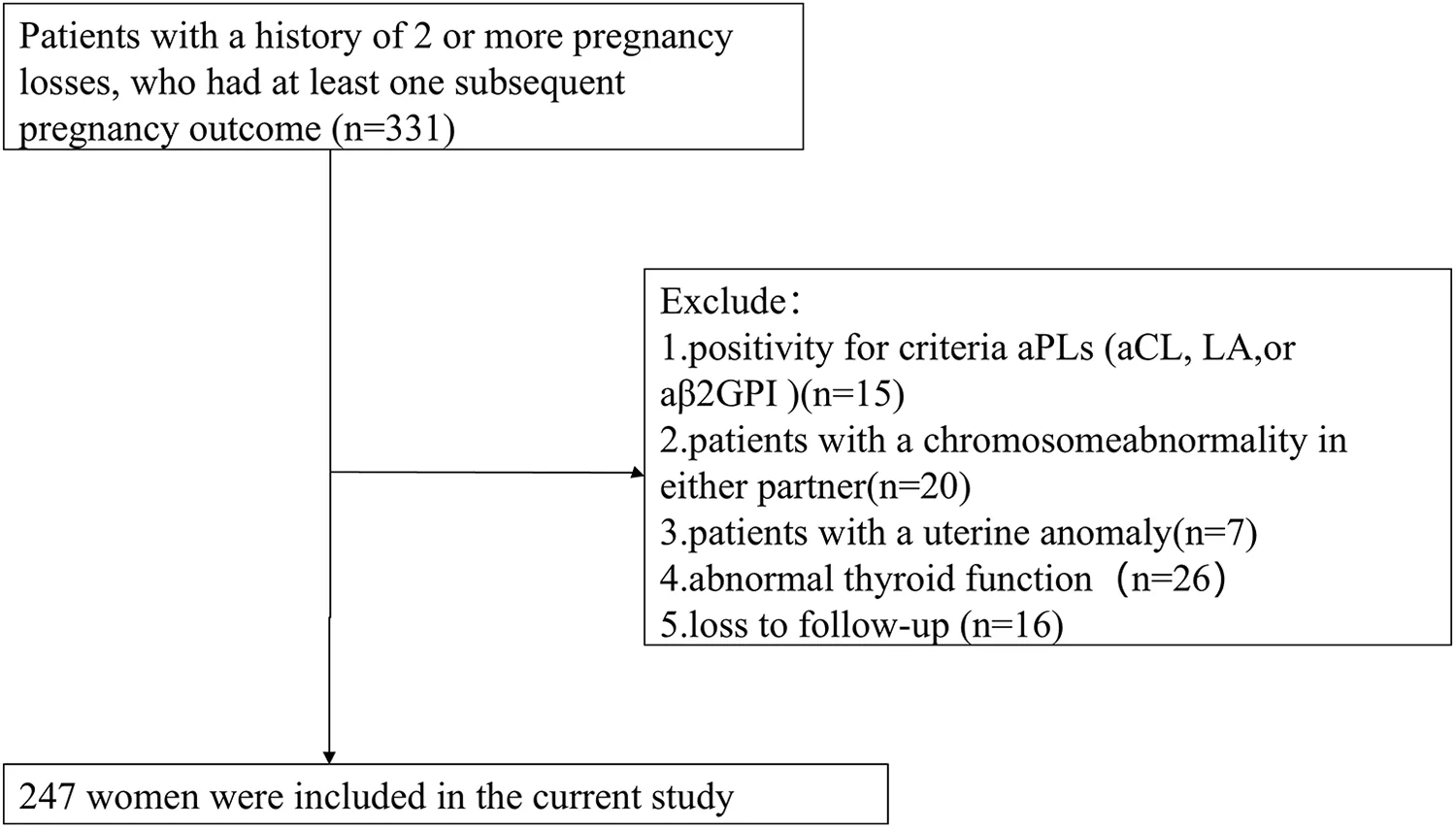
Flow chart illustrating recruitment of subjects.

### 2.2 Antibody and laboratory measurement

Blood samples for criteria aPLs and NC-aPLs testing were collected at the first visit, which occurred either before conception or before 7 weeks of gestation. All NC-aPLs (aPE, aPT, aAnxA5, aPS, aPA, and aPI) were tested for both IgG and IgM isotypes using a line immunoassay (LIA) according to the manufacturer's guidelines (GA Generic Assays GmbH, Dahlewitz, Germany). Briefly, patient serum samples were applied to polyvinylidene difluoride (PVDF) membranes, which were coated with phospholipids arranged in lines on a strip. These strips were subsequently analyzed using densitometry with a scanner equipped with Dot Blot Analyzer software (GA Generic Assays GmbH). An optical density value equal to or exceeding 50 was considered indicative of a positive result. Furthermore, aPS/PT IgG and IgM were quantified through an enzyme-linked immunosorbent assay (ELISA) using specific kits (HUMAN Diagnostics, Germany), adhering to the manufacturer's protocol. The threshold value for aPS/PT was established at 30 U/mL. In accordance with prior research, the positivity of NC-aPLs was determined by the presence of at least one NC-aPLs from the panel tested (12). It is posited that NC-aPLs contribute to the overall immunological profile rather than functioning independently as individual antibodies (13).

Criteria aPLs, including LA, aCL, and aβ2GPI, were assessed. The LA assays were conducted in accordance with the guidelines established by the International Society on Thrombosis and Haemostasis (ISTH), incorporating screening tests, mixing studies, and confirmatory tests, with a positive result indicated by a screen/confirm time ratio exceeding 1.20. IgG, IgM, and IgA isotypes of aCL and aβ2GPI were quantified in serum using chemiluminescent immunoassay (CLIA), with positivity thresholds set at 10 U/mL and 20 U/mL, respectively. Patients who tested positive for any of the criteria aPLs were excluded from the study. Repeat testing at 12 weeks was not conducted.

### 2.3 Treatment strategy

All patients were administered dydrogesterone (10 mg three times daily) as baseline luteal support from the confirmation of pregnancy until either pregnancy loss or the completion of 12 weeks of gestation. Subsequently, antithrombotic and immunomodulatory therapies were tailored based on individual risk profiles, which included NC-aPLs, previous pregnancy history, and thrombosis. The treatment regimens comprised: (1) low-dose aspirin (LDA, 50–100 mg/day), (2) LDA combined with low-molecular-weight heparin (LMWH), and (3) the addition of hydroxychloroquine (HCQ, 0.2 g twice daily) and/or prednisone (10 mg/day) for patients with high-risk features, such as three or more previous miscarriages, prior late pregnancy loss, stillbirth, severe preeclampsia, fetal growth restriction, preterm birth, or recurrent pregnancy loss despite standardized etiological treatment. After aPLs results were available, LDA, LMWH, and HCQ were started either before conception or after pregnancy confirmation, and prednisone after pregnancy confirmation. LDA was continued until 36 weeks of gestation, LMWH and HCQ until delivery or per clinical judgment, and prednisone until 12 weeks.

### 2.4 Outcome measures

APO were categorized as follows: (1) pregnancy loss, which included biochemical pregnancy loss (characterized by a positive urinary human chorionic gonadotropin test without ultrasound confirmation of a gestational sac), early miscarriage (clinical pregnancy up to 10 weeks of gestation), and late miscarriage (10–24 weeks of gestation); (2) stillbirth, characterized by fetal delivery without signs of life at or beyond 24 completed weeks of gestation (14); (3) preterm delivery, defined as occurring before 37 weeks of gestation due to conditions such as pre-eclampsia, eclampsia, fetal growth restriction (FGR), and/or placental insufficiency; (4) FGR, identified as estimated fetal weight below the 10th percentile for gestational age; (5) pre-eclampsia, defined as new-onset persistent hypertension (blood pressure >140/90 mmHg) after 20 weeks of gestation, with either new-onset proteinuria (>300 mg/24 h) or organ dysfunction; and (6) intrauterine distress, defined as fetal heart rate <110 or >160 beats per minute, accompanied by meconium-stained amniotic fluid, abnormal fetal movements, and Umbilical Artery pH <7.2.

Other pregnancy complications, including intrahepatic cholestasis of pregnancy (ICP), premature rupture of membranes (PROM), gestational diabetes mellitus (GDM), placenta previa, placental abruption, and gestational hypertension, were retrieved from the electronic medical record system of the medical records department.

### 2.5 Statistical analysis

Categorical variables were described using frequencies and percentages, and intergroup comparisons were conducted utilizing the chi-square test. Univariable logistic regression analysis was employed to estimate odds ratios (OR) with corresponding 95% confidence intervals (CI). Continuous variables were characterized by the mean ± standard deviation (SD) for data exhibiting a normal distribution, or by the median and interquartile range (IQR)for data not conforming to a normal distribution. For normally distributed continuous variables, Cohen's d and its 95% CI were calculated using an independent samples t-test. In contrast, for non-normally distributed data, the effect size r was determined as r = Z/√N, with the 95% CI derived through Fisher's Z transformation, employing the Mann–Whitney U test. Univariate analysis was employed to identify potential candidate variables (*P* < 0.05) for inclusion in binary logistic regression model. Baseline confounding factors, including maternal age, body mass index (BMI), history of prior labor abortion, number of previous pregnancy losses, pregnancy history, and *in vitro* fertilization-embryo transfer (IVF-ET), were incorporated into the models irrespective of their significance in the univariate analysis. Additionally, NC-aPLs positivity, the primary exposure of interest, was consistently retained in the model. All statistical analyses were conducted using IBM SPSS Statistics (Version 29.0), with a two-tailed *P*-value of less than 0.05 considered statistically significant.

## 3 Results

### 3.1 Positive rates of the non-criteria antiphospholipid antibodies

The positivity rates of the evaluated NC-aPLs are presented in Table 1. Within the cohort of 247 patients with RPL, the most frequently identified antibody was aPE IgM, detected in 14.17% of the patients. This was followed by aPT IgM at 11.74%, aAnxA5 IgM at 8.50%, aAnxA5 IgG at 6.07%, and aPS/PT IgM at 5.26%. No positive results were observed for aPS/PT IgG or aPA IgM. Regarding the co-occurrence of antibodies, one patient was positive for four NC-aPLs subtypes, four patients were positive for three subtypes, and twenty-seven patients were positive for two subtypes.

**Table 1:** Positive rates of the non-criteria antiphospholipid antibodies in recurrent pregnancy loss patients.

NC-aPLs	Positive rates (n/%)
aPE-IgM	35 (14.17%)
aPT-IgM	29 (11.74%)
aAnxA5-IgM	21 (8.50%)
aAnxA5-IgG	15 (6.07%)
aPS/PT-IgM	13 (5.26%)
aPI-IgG	4 (1.62%)
aPS-IgG	4 (1.62%)
aPA-IgG	3 (1.21%)
aPI-IgM	3 (1.21%)
aPT-IgG	3 (1.21%)
aPS-IgM	2 (0.81%)
aPE-IgG	1 (0.40%)

aPE,anti-phosphatidylethanolamine; aPT, anti-prothrombin; aAnxA5, anti-annexin A5; aPS/PT,anti-phosphatidylserine/prothrombin; aPI,anti-phosphatidylinositol;aPS,anti-phosphatidylserine; aPA,anti-phosphatidic acid.

### 3.2 Demographic characteristics

Among the 247 patients studied, 95 individuals (38.46%) tested positive for at least one NC-aPLs, while 152 individuals (61.54%) tested negative for all NC-aPLs. Within the NC-aPLs-positive cohort, the incidence of women with three or more prior miscarriages was significantly higher compared to the NC-aPLs-negative cohort (38.95% vs. 23.68%, *p* = 0.011). Additionally, significant differences were observed between the two groups regarding treatment modalities, including the use of low molecular weight heparin (LMWH) (86.31% vs. 74.34%, *p* = 0.039), glucocorticoids (26.32% vs. 14.47%, *p* = 0.021), and hydroxychloroquine (HCQ) (82.11% vs. 62.50%, *p* < 0.001). No significant differences were identified between the groups concerning maternal age, BMI, obesity, utilization of IVF-ET, pregnancy history, or previous labor abortion (*p* > 0.05). A comprehensive summary of the baseline demographic and clinical characteristics of both cohorts is provided in Table 2.

**Table 2:** Demographic characteristics of participants in different patient groups.

Characteristics	NC-aPLs (-)	NC-aPLs (+)	U or *χ*^2^	*P*-value
	*n* = 152	*n* = 95		
Age (years)	31.00 (28.00, 35.00)	30.00 (28.00, 33.00)	6607	0.260
BMI (kg/m2)	21.81 (20.34, 23.99)	21.48 (19.83, 23.62)	6468	0.169
Obesity	4 (2.63)	1 (1.05)	0.735	0.652
Hypertension	1 (0.66)	0	——	1.000^a^
Smoke	2 (1.32)	1 (1.05)	——	1.000^a^
History of thrombosis	1 (0.66)	1 (1.05)	——	1.000^a^
Number of previous miscarriages			6.542	0.011*
2	116 (76.32)	58 (61.05)		
3 or more	36 (23.68)	37 (38.95)		
History of biochemical pregnancy losses	49 (32.24)	32 (33.68)	0.056	0.814
History of early miscarriage	135 (88.82)	90 (94.74)	2.526	0.112
History of late miscarriage	20 (13.16)	13 (13.68)	0.014	0.906
History of stillbirth	7 (4.61)	1 (1.05)	2.354	0.158
Previous preeclampsia	1 (0.66)	0	——	1.000^a^
Previous premature delivery	3 (1.97)	1 (1.05)	——	1.000^a^
Previous FGR	2 (1.32)	1 (1.05)	——	1.000^a^
Pregnancy history			2.943	0.086
Nulliparous	114 (75.00)	80 (84.21)		
Mulliparous	38 (25.00)	15 (15.79)		
Previous labor abortion	50 (32.89)	24 (25.26)	1.623	0.203
IVF-ET	11 (7.38)	7 (7.61)	0.004	0.948
Treatment during pregnancy	128 (84.21)	84 (88.42)	0.852	0.356
LDA			1.376	0.503
Never	25 (16.45)	11 (11.58)		
Before pregnancy	62 (40.79)	38 (40.00)		
After pregnancy	65 (42.76)	46 (48.42)		
LWMH			6.469	0.039*
Never	39 (25.66)	13 (13.68)		
Before pregnancy	0	1 (1.05)		
After pregnancy	113 (74.34)	81 (85.26)		
Glucocorticoid			5.321	0.021*
Never	130 (85.53)	70 (73.68)		
After pregnancy	22 (14.47)	25 (26.32)		
HCQ			43.307	＜0.001***
Never	57 (37.50)	17 (17.89)		
Before pregnancy	10 (6.58)	38 (40.00)		
After pregnancy	85 (55.92)	40 (42.11)		

The data shown are median (interquartile range), *n* (%).

BMI, body mass index; IVF-ET, *in vitro* fertilization-embryo transfer; LDA, low-dose aspirin; LWMH, low molecular weight heparin; HCQ, hydroxychloroquine.

a *P*-value was calculated using Fisher's exact test (2-tailed) due to expected cell counts < 5 in 50% of the cells.

* *p* < 0.05.

*** *p* < 0.001.

### 3.3 Association of NC-aPLs with pregnancy outcomes and complications

Table 3 presents the pregnancy outcomes and complications among patients with RPL, categorized by the status of NC-aPLs. The overall incidence of APO was similar between the NC-aPLs-negative and NC-aPLs-positive groups (46.71% vs. 53.68%, OR = 1.322, 95% CI 0.791–2.211, *P* = 0.286). Similarly, no significant differences were observed between the groups in terms of pregnancy loss rates (40.79% vs. 42.11%, OR = 1.056, 95% CI 0.628–1.776, *P* = 0.838) or other APO and complications, with the exception of neonatal weight, which was significantly lower in the NC-aPLs-positive group (median neonatal weight: 3,000.00 g vs. 3,170.00 g, r = 0.329, 95% CI −0.023–0.681, *P* = 0.017; median neonatal weight z-score: −0.39 vs. 0.11, r = 0.193, 95% CI 0.026–0.349, *P* = 0.024). Subgroup analyses showed no significant associations between individual NC-aPLs subtypes (aPE IgM, aPT IgM, or aAnxA5 IgM) and APO or complications (Table 4). Furthermore, the incidence of APO and complications did not significantly differ between the multiple positive NC-aPLs subgroup and the single positive NC-aPLs subgroup (Table 5).

**Table 3:** Pregnancy outcomes and complications among patients with recurrent pregnancy loss who were NC-aPLs positive or negative.

Outcomes	NC-aPLs(-)	NC-aPLs(+)	U or χ^2^	*P*-value	Effect Size (95% CI)^a^
	*n* = 152	*n* = 95			
Overall APO	71 (46.71)	51 (53.68)	1.137	0.286	1.322 (0.791–2.211)
Total pregnancy loss	62 (40.79)	40 (42.11)	0.042	0.838	1.056 (0.628–1.776)
Chemical pregnancy	9 (5.92)	10 (10.53)	1.746	0.186	0.535 (0.209–1.369)
Early miscarriage	49 (32.24)	28 (29.47)	0.208	0.648	1.138 (0.652–1.987)
Late miscarriage	4 (2.63)	2 (2.11)	——	1.000^b^	1.257 (0.226–6.998)
Stillbirth	1 (0.66)	0	——	1.000^b^	——
Preterm delivery	2 (1.32)	6 (6.32)	4.664	0.058	0.198 (0.039–1.001)
Pre-eclampsia	3 (1.97)	4 (4.21)	1.062	0.434	0.458 (0.100–2.093)
FGR	2 (1.32)	2 (2.11)	0.229	0.640	0.620 (0.086–4.477)
Intrauterine distress	3 (1.97)	4 (4.21)	1.062	0.434	0.458 (0.100–2.093)
GDM	9 (5.92)	2 (2.11)	2.000	0.272	0.342 (0.072–1.617)
ICP	3 (1.97)	1 (1.05)	——	1.000^b^	0.528 (0.054–5.155)
Placenta abruption	1 (0.66)	2 (2.11)	1.021	0.561	3.247 (0.290–36.312)
Placenta previa	2 (1.32)	1 (1.05)	——	1.000^b^	0.798 (0.071–8.921)
Gestational hypertension	2 (1.32)	1 (1.05)	——	1.000^b^	0.798 (0.071–8.921)
PROM	15 (9.87)	7 (7.37)	0.450	0.502	0.727 (0.285–1.853)
Neonatal weight (g)	3170.00 (2935.00, 302.50)	3000.00 (2720.00, 3200.00)	1576.000	0.017*	0.329 (−0.023–0.681)
Neonatal weight Z-score^c^	0.11 (−0.47, 0.46)	−0.39 (−0.90, 0.29)	1716.000	0.024*	0.193 (0.026–0.349)

The data shown are median (interquartile range), *n* (%).

APO, adverse pregnancy outcomes; FGR, fetal growth restriction; GDM, gestational diabetes mellitus; ICP, intrahepatic cholestasis of pregnancy; PROM, premature rupture of membranes.

a 95% CI of OR for categorical variables, 95% CI of r for Neonatal weight (g) and Birth weight Z-score (Mann–Whitney U test).

b *P*-value was calculated using Fisher's exact test (2-tailed) due to expected cell counts < 5 in 50% of the cells.

c birthweight Z-scores was calculated by using the INTERGROWTH-21st newborn growth standards, which adjust for gestational age and sex.

* *p* < 0.05.

**Table 4:** Pregnancy outcomes and complications stratified by positivity for aPE IgM, aPT IgM, and anti-AnxA5 IgM.

Outcomes	aPE-IgM		aAnxA5-IgM		aPT-IgM	
	P	Effect Size (95% CI)^a^	P	Effect Size (95% CI)^a^	P	Effect Size (95% CI)^a^
Overall APO	0.916	0.962 (0.471–1.968)	0.531	0.750 (0.304–1.850)	0.508	1.300 (0.597–2.833)
Total pregnancy loss	0.867	0.939 (0.453–1.948)	0.879	1.073 (0.434–2.649)	0.992	1.004 (0.457–2.205)
Chemical pregnancy	0.739	1.148 (0.317–4.166)	0.669	1.294 (0.278–6.028)	0.254	2.165 (0.666–7.037)
Early miscarriage	0.720	0.866 (0.393–1.904)	0.823	1.114 (0.431–2.881)	0.384	0.673 (0.274–1.649)
Late miscarriage	1.000^b^	1.218 (0.138–10.744)	1.000^b^	——	0.531	1.521 (0.171–13.498)
Stillbirth	1.000^b^	——	1.000^b^	——	1.000^b^	——
Preterm delivery	0.316	2.081 (0.403–10.748)	1.000^b^	——	0.055	4.915 (1.110–21.770)
Pre-eclampsia	0.598	——	1.000^b^	——	0.588	1.262 (0.146–10.870)
FGR	1.000^b^	——	1.000^b^	——	0.069	8.000 (1.082–59.135)
Intrauterine distress	1.000^b^	1.010 (0.118–8.651)	1.000^b^	——	0.588	1.262 (0.146–10.870)
GDM	0.372	——	0.606	——	1.000^b^	0.743 (0.092–6.025)
ICP	0.459	2.049 (0.207–20.275)	0.301	3.717 (0.369–37.404)	1.000^b^	——
Placenta abruption	1.000^b^	——	0.235	5.600 (0.486–64.484)	0.314	3.857 (0.339–43.924)
Placenta previa	0.369	3.088 (0.273–34.996)	1.000^b^	——	1.000^b^	——
Gestational hypertension	0.369	3.088 (0.273–34.996)	1.000^b^	——	1.000^b^	——
PROM	1.000^b^	0.952 (0.266–3.404)	0.703	0.488 (0.062–3.822)	0.486	0.335 (0.043–2.589)
Neonatal weight (g)	0.368	−0.147 (−0.506–0.212)	0.440	0.293 (−0.154–0.740)	0.043*	−0.543 (−0.933 to −0.153)
Neonatal weight Z-score ^c^	0.530	−0.102 (−0.461–0.257)	0.467	0.331 (−0.116–0.778)	0.073	−0.472 (−0.862 to −0.082)

The data shown are median (interquartile range), *n* (%).

aPE, anti-phosphatidylethanolamine; aPT, anti-prothrombin; aAnxA5, anti-annexin A5; APO, adverse pregnancy outcomes; FGR, fetal growth restriction; GDM, gestational diabetes mellitus; ICP, intrahepatic cholestasis of pregnancy; PROM, premature rupture of membranes.

a 95% CI of OR for categorical variables, 95% CI of r for Neonatal weight (g) and Birth weight Z-score (Mann–Whitney U test).

b *P*-value was calculated using Fisher's exact test (2-tailed) due to expected cell counts < 5 in 50% of the cells.

c birthweight Z-scores was calculated by using the INTERGROWTH-21st newborn growth standards, which adjust for gestational age and sex.

*p < 0.05.

**Table 5:** Pregnancy outcomes and complications in different NC-aPLs status.

Outcomes	Single Positive *n* = 63	Multiple Positive *n* = 32	U or χ^2^	*P*-value	Effect Size (95% CI)^a^
Overall APO	32 (50.79)	19 (59.38)	0.628	0.428	1.416 (0.598–3.350)
Total pregnancy loss	26 (41.27)	14 (43.75)	0.054	0.817	1.107 (0.468–2.615)
Chemical pregnancy	5 (7.94)	5 (15.63)	0.332	0.297	2.148 (0.573–8.050)
Early miscarriage	19 (30.16)	9 (28.13)	0.042	0.837	0.906 (0.354–2.319)
Late miscarriage	2 (3.17)	0	1.038	0.548	——
Stillbirth	0	0	——	——	——
Preterm delivery	4 (6.35)	2 (6.25)	——	1.000^b^	0.9830.170–5.678)
Pre-eclampsia	2 (3.17)	2 (6.25)	0.498	0.601	2.033（0.273–15.147）
FGR	1 (1.59)	1 (3.13)	——	1.000^b^	2.000 (0.121–33.059)
Intrauterine distress	3 (4.76)	1 (3.13)	——	1.000^b^	0.645（0.064–6.463）
GDM	1 (1.59)	1 (3.13)	——	1.000^b^	2.000 (0.121–33.059)
ICP	0	1 (3.13)	1.990	0.337	——
Placenta abruption	1 (1.59)	1 (3.13)	——	1.000^b^	2.000 (0.121–33.059)
Placenta previa	0	1 (3.13)	1.990	0.337	——
Gestational hypertension	1 (1.59)	0	——	1.000^b^	——
PROM	5 (7.94)	2 (6.25)	——	1.000^b^	0.773 (0.142–4.225)
Neonatal weight (g)	3060.00 (2800.00, 3300.00)	2830.00 (2572.50, 3035.00)	182.000	0.047*	0.563 (0.130–0.996)
Neonatal weight Z-score^c^	−0.39 (−0.84, 0.34)	−0.37 (−1.00, 0.22)	289.000	0.625	0.222 (−0.205–0.649)

The data shown are median (interquartile range), *n* (%).

APO, adverse pregnancy outcomes; FGR, fetal growth restriction; GDM, gestational diabetes mellitus; ICP, intrahepatic cholestasis of pregnancy; PROM, premature rupture of membranes.

a 95% CI of OR for categorical variables, 95% CI of r for Neonatal weight (g) and Birth weight Z-score (Mann–Whitney U test).

b *P*-value was calculated using Fisher's exact test (2-tailed) due to expected cell counts < 5 in 50% of the cells.

c birthweight Z-scores was calculated by using the INTERGROWTH-21st newborn growth standards, which adjust for gestational age and sex.

*p < 0.05.

### 3.4 Analysis of the factors associated with adverse pregnancy outcomes

Among the 247 pregnancies analyzed, 122 resulted in APO. Univariate analysis identified significant associations between APO and several factors, including GDM, PROM, treatment during pregnancy, use of LDA, LMWH and HCQ (Table 6). In binary logistic regression analysis, after adjusting for potential confounders, the intensity of medication was associated with lower odds of APO. Compared to patients who received no treatment, those administered 1–2 types of medications had significantly lower odds of APO (aOR = 0.177, 95% CI 0.055–0.569, *P* = 0.004). This association was even stronger in patients receiving 3–4 types of medications (aOR = 0.102, 95% CI 0.037–0.283, *P* < 0.001). In contrast, neither single (aOR = 1.553, 95% CI 0.799–3.017, *P* = 0.194) nor multiple NC-aPLs positivity (aOR = 1.942, 95% CI 0.798–4.726, *P* = 0.144) achieved statistical significance (Table 7). Stratified analyses showed that, compared with no treatment or low-intensity therapy, escalated therapy was associated with lower odds of overall APO in the NC-aPLs-positive group (OR = 0.214, 95% CI 0.057–0.809, *P* = 0.015; Table 8). Similar findings were observed in the NC-aPLs-negative group (Table 9).

**Table 6:** Univariate comparison of factors between women with and without adverse pregnancy outcomes.

Characteristics	No adverse pregnancies	Adverse regnancies	U or χ^2^	*P*-value
	*n* = 125	*n* = 122		
Age (years)	31.00 (28.00, 34.00)	31.00 (28.75, 34.00)	8165.000	0.335
BMI (kg/m^2^)	21.87 (19.98, 23.85)	21.44 (20.06, 23.86)	7634.500	0.986
Obesity	3 (2.40)	2 (1.64)	——	1.000^a^
Hypertension	0	1 (0.82)	1.029	0.494
Smoke	0	3 (2.46)	3.112	0.119
History of thrombosis	1 (0.80)	1 (0.82)	——	1.000^a^
Number of previous miscarriages			0.087	0.768
2	87 (69.60)	87 (71.31)		
3 or more	38 (30.40)	35 (28.69)		
History of biochemical pregnancy losses	36 (28.80)	45 (36.89)	1.831	0.176
History of early miscarriage	118 (94.40)	107 (87.70)	3.411	0.065
History of late miscarriage	17 (13.60)	16 (13.11)	0.013	0.911
History of stillbirth	4 (3.20)	4 (3.28)	——	1.000^a^
Previous preeclampsia	0	1 (0.82)	1.029	0.494
Previous premature delivery	1 (0.80)	3 (2.46)	1.067	0.366
Previous FGR	2 (1.60)	1 (0.82)	——	1.000^a^
Pregnancy history			0.133	0.715
Nulliparous	97 (77.6)	97 (79.5)		
Mulliparous	28 (22.4)	25 (20.5)		
Previous labor abortion	37 (29.60)	37 (30.33)	0.016	0.901
IVF-ET	7 (5.74)	11 (9.24)	1.071	0.301
Treatment during pregnancy	120 (96.00)	92 (75.41)	21.522	＜0.001***
LDA			22.788	＜0.001***
Never	5 (4.00)	31 (25.41)		
Before pregnancy	64 (51.20)	47 (38.52)		
After pregnancy	56 (44.80)	44 (36.07)		
LWMH			21.322	＜0.001***
Never	12 (9.60)	40 (32.79)		
Before pregnancy	113 (90.40)	81 (66.39)		
After pregnancy	0	1 (0.82)		
Glucocorticoid			0.065	0.799
Never	102 (81.60)	98 (80.33)		
After pregnancy	23 (18.40)	24 (19.67)		
HCQ	0	0		
Never			11.506	0.003**
Before pregnancy	26 (20.80)	48 (39.34)		
After pregnancy	75 (60.00)	50 (40.98)		
GDM	10 (8.00)	1 (0.82)	7.481	0.006**
ICP	4 (3.20)	0	3.968	0.122
Placenta abruption	1 (0.80)	2 (1.64)	0.363	0.619
Placenta previa	1 (0.80)	2 (1.64)	0.363	0.619
Gestational hypertension	2 (1.60)	1 (0.82)	——	1.000^a^
PROM	20 (16.00)	2 (1.64)	15.693	＜0.001***
NC-aPLs positive number			1.763	0.414
0	81 (64.80)	71 (58.20)		
Single positive	31 (24.80)	32 (26.23)		
Multiple Positive	13 (10.40)	19 (15.57)		

The data shown are median (interquartile range), *n* (%).

BMI, body mass index; FGR, fetal growth restriction; IVF-ET, *in vitro* fertilization-embryo transfer; LDA, low-dose aspirin; LWMH, low molecular weight heparin; HCQ, hydroxychloroquine; GDM, gestational diabetes mellitus; ICP, intrahepatic cholestasis of pregnancy; PROM, premature rupture of membranes; NC-aPLs, non-criteria antiphospholipid antibodies.

a *P*-value was calculated using Fisher's exact test (2-tailed) due to expected cell counts < 5 in 50% of the cells.

**p < 0.01，***p < 0.001.

**Table 7:** Multivariable logistic regression analyses of factors associated with adverse pregnancy outcomes.

Variables	β	S.E.	Wald χ^2^	P	OR (95% CI)
Age	0.065	0.038	2.933	0.087	1.067 (0.991–1.150)
BMI	0.028	0.042	0.444	0.505	1.029 (0.947–1.117)
Prior labor abortion (yes v.s. no)	−0.050	0.324	0.024	0.877	0.951 (0.504–1.794)
Prior pregnancy loss number (3 times v.s. twice)	−0.050	0.316	0.025	0.875	0.952 (0.512–1.767)
Pregnancy history (Nulliparous v.s. Mulliparous)	−0.285	0.391	0.533	0.465	0.752 (0.349–1.617)
IVF-ET (yes v.s. no)	0.466	0.544	0.736	0.391	1.594 (0.549–4.626)
NC-aPLs (Ref as negative)			3.002	0.223	
Single positive	0.440	0.339	1.688	0.194	1.553 (0.799–3.017)
Multiple positive	0.664	0.454	2.138	0.144	1.942 (0.798–4.726)
Medicinal degree (Ref as none medicine)			19.648	＜0.001***	
1 or 2 types of medicine	−1.733	0.597	8.441	0.004**	0.177 (0.055–0.569)
3 or 4 types of medicine	−2.285	0.521	19.214	＜0.001***	0.102 (0.037–0.283)

BMI, body mass index; IVF-ET, *in vitro* fertilization-embryo transfer; NC-aPLs, non-criteria antiphospholipid antibodies.

Model adjusted for age, BMI, history of abortion, prior pregnancy loss number, pregnancy history, and IVF-ET. Hosmer-Lemeshow test: χ^2^ = 18.248, *P* = 0.019. Nagelkerke R^2^ = 0.170.

**p < 0.01.

***p < 0.001.

**Table 8:** Association of medication intensity with pregnancy outcomes and complications in NC-aPLs-positive groups.

Outcomes	Low therapy or non-Treat	Escalated therapy	U or χ^2^	*P*-value	Effect Size (95% CI)^a^
Overall APO	13 (25.49)	38 (74.51)	5.88	0.015*	0.214 (0.057–0.809)
Total pregnancy loss	13 (32.50)	27 (67.50)	12.094	<0.001***	0.120 (0.031–0.457)
Chemical pregnancy	7 (70.00)	3 (30.00)	22.550	<0.001***	0.051 (0.011–0.232)
Early miscarriage	6 (21.43)	22 (78.57)	0.596	0.549	0.643 (0.209–1.982)
Late miscarriage	0	2	——	1.000^b^	——
Stillbirth	0	0	——	——	——
Preterm delivery	0	6	——	1.000^b^	——
Pre-eclampsia	0	4	——	1.000^b^	——
FGR	0	2	——	1.000^b^	——
Intrauterine distress	0	4	——	1.000^b^	——
GDM	0	2	——	1.000^b^	——
ICP	0	1	——	1.000^b^	——
Placenta abruption	0	2	——	1.000^b^	——
Placenta previa	0	1	——	1.000^b^	——
Gestational hypertension	0	1	——	1.000^b^	——
PROM	0	7	——	1.000^b^	——
Neonatal weight (g)	3475.00 (3350.00, 3475.00)	2980.00 (2715.00, 3165.00)	10.000	0.058	——
Neonatal weight Z-score^c^	0.41 (0.06, 0.41)	−0.40 (−0.96, 0.26)	22.000	0.176	——

The data shown are median (interquartile range), *n* (%).

APO, adverse pregnancy outcomes; FGR, fetal growth restriction; GDM, gestational diabetes mellitus; ICP, intrahepatic cholestasis of pregnancy; PROM, premature rupture of membranes.

a 95% CI of OR for categorical variables, 95% CI of r for Neonatal weight (g) and Birth weight Z-score (Mann–Whitney U test).

b *P*-value was calculated using Fisher's exact test (2-tailed) due to expected cell counts < 5 in 50% of the cells.

c birthweight Z-scores was calculated by using the INTERGROWTH-21st newborn growth standards, which adjust for gestational age and sex.

*p < 0.05.

*** < 0.001.

**Table 9:** Association of medication intensity with pregnancy outcomes and complications in NC-aPLs-negative groups.

Outcomes	Off therapy	On therapy	U or χ^2^	*P*-value	Effect Size (95% CI)^a^
Overall APO	20 (28.17)	51 (71.83)	15.356	<0.001***	0.132 (0.043–0.410)
Total pregnancy loss	20 (32.26)	42 (67.74)	21.359	<0.001***	0.098 (0.031–0.304)
Chemical pregnancy	3 (33.33)	6 (66.67)	2.214	0.152	0.344 (0.080–1.484)
Early miscarriage	15 (30.61)	34 (69.39)	11.949	<0.001***	0.217 (0.087–0.542)
Late miscarriage	2 (50.00)	2 (50.00)	3.616	0.118	0.175 (0.023–1.305)
Stillbirth	0	1	——	1.000^b^	——
Preterm delivery	0	2	——	1.000^b^	——
Pre-eclampsia	0	3	——	1.000^b^	——
FGR	0	2	——	1.000^b^	——
Intrauterine distress	0	3	——	1.000^b^	——
GDM	0	9	1.794	0.356	——
ICP	0	3	——	1.000^b^	——
Placenta abruption	1	0	——	1.000^b^	——
Placenta previa	0	2	——	1.000^b^	——
Gestational hypertension	1	1	——	1.000^b^	——
PROM	0	15	3.120	0.130	——
Neonatal weight (g)	3240.00 (3075.00, 3300.00)	3170.00 (2917.50, 3317.50)	127.500	0.539	——
Neonatal weight Z-score ^c^	0.02 (−0.60, 0.45)	0.13 (−0.47, 0.46)	175.500	0.745	——

The data shown are median (interquartile range), *n* (%).

Abbreviations:APO,adverse pregnancy outcomes;FGR,fetal growth restriction;GDM,gestational diabetes mellitus;ICP,intrahepatic cholestasis of pregnancy;PROM,premature rupture of membranes.

a 95% CI of OR for categorical variables, 95% CI of r for Neonatal weight (g) and Birth weight Z-score (Mann–Whitney U test).

b *P*-value was calculated using Fisher's exact test (2-tailed) due to expected cell counts < 5 in 50% of the cells.

c birthweight Z-scores was calculated by using the INTERGROWTH-21st newborn growth standards, which adjust for gestational age and sex.

*** < 0.001.

## 4 Discussion

This study examined the prevalence of NC-aPLs in patients with RPL and their association with APO. Although NC-aPLs were detectable in a substantial proportion of RPL patients, the overall incidence of APO did not significantly differ between NC-aPLs-positive and NC-aPLs-negative groups. Notably, the NC-aPLs -positive cohort received significantly more intensive interventions, including HCQ, LMWH, and glucocorticoids. Univariate analyses identified GDM, PROM, treatment during pregnancy, use of LDA, LMWH, and HCQ as factors associated with APO. However, multivariable regression analysis showed that NC-aPLs were not associated with APO, whereas treatment intensity was associated with lower odds of APO. Nonetheless, these findings provide a reference for future research to elucidate the potential clinical significance of NC-aPLs.

The detection rates of NC-aPLs in our cohort is 38.46%, with aPE-IgM (14.17%), aPT-IgM (11.74%), and aAnxA5-IgM (8.50%) being the most prevalent. These findings align with previous studies, although exact prevalence rates differ across studies. For example, Zhu et al. reported a higher prevalence of aPE-IgM (40.0%) and aPT-IgM (10.4%) in Chinese RPL patients (15), while Mu et al. identified aAnxA5 as the most common specificity (10.1%), followed by aPC (9.2%), aPE (7.4%) and aPT IgG (7.4%) (16). These discrepancies may be attributed to variations in study design, detection platforms, antibody cut-off values, and laboratory standards.

Our study indicated that the NC-aPLs-positive cohort exhibited a significantly higher proportion of women with a history of three or more prior miscarriages compared to the NC-aPLs-negative cohort (38.95% vs. 23.68%, *P* = 0.011), aligning with the findings reported by Finan et al. and Xiang et al. (17, 18). To address this disparity, the number of previous pregnancy losses was incorporated as a covariate in the multivariable analysis. Except for treatment, the baseline characteristics were otherwise comparable between the two groups within our study cohort.

GDM and PROM were found to be significantly correlated with APO. It is noteworthy that previous studies have identified associations between NC-aPLs and these complications. For instance, Liu et al. reported a GDM incidence rate of 22.7% among patients with non-criteria obstetric APS (19), which surpasses the 15.6% prevalence observed in the general pregnant population in China (20). Additionally, a retrospective study involving 408 women with miscarriage identified aPS/PT IgG and IgM as independent risk factors for PROM. Collectively, this body of evidence implies that GDM and PROM may act as intermediate variables within the causal pathway linking NC-aPLs to APO (21). Consequently, we excluded these variables from the binary logistic regression model to mitigate overadjustment bias.

Pregnancy loss rates were comparable between the NC-aPLs positive and NC-aPLs-negative groups (42.11% vs. 40.79%, OR = 1.056, 95% CI 0.628–1.776, *P* = 0.838), consistent with the findings of Mu et al. (13). However, the overall miscarriage rate observed in our cohort was higher than that reported in previous studies. This discrepancy may be attributed to two primary factors. Firstly, our definition of pregnancy loss encompassed biochemical pregnancies, whereas most prior studies limited their analyses to ultrasound-confirmed clinical miscarriages. For example, Kim et al. reported a miscarriage rate of only 26.5% using the latter definition (22). Secondly, a significant proportion of our participants had a history of three or more prior pregnancy losses. As demonstrated by Ogasawara et al., the risk of miscarriage increases progressively with the number of previous losses: 23.2% in women with two prior losses, 32.4% in those with three, and over 37% in those with four or more (23).

Other APO, including stillbirth, preeclampsia, FGR, preterm birth, and intrauterine distress, also did not differ significantly between groups. These findings align with those of Xiang et al., who reported that neither aPT nor aPS/PT was associated with preterm delivery, FGR, or preeclampsia (24). Nonetheless, the clinical utility of NC-aPLs remains a topic of considerable debate. Some studies have advocated for the inclusion of NC-aPLs in the diagnostic evaluation of seronegative APS, suggesting that their detection may aid in risk stratification and timely intervention to prevent APO (18, 25, 26). Conversely, a 2024 review underscored that inter-patient heterogeneity in clinical presentation and the lack of standardized assays continue to hinder the routine application of NC-aPLs testing (27). Moreover, NC-aPLs have not been incorporated into the 2023 ACR/EULAR APS classification criteria. Therefore, larger prospective studies with standardized assays are needed to clarify their role.

Our study found a modest yet statistically significant reduction in neonatal weight among the NC-aPLs-positive group (3,000.00 g vs. 3,170.00 g, r = 0.329, 95% CI −0.023–0.681, *P* = 0.017). This difference persisted after adjusting for gestational age and sex using birth weight z-scores (−0.39 vs. 0.11, r = 0.193, 95% CI 0.026–0.349, *P* = 0.024). Nevertheless, the effect size was small, and subgroup analyses based on antibody subtype and single versus multiple positivity did not yield significant differences. Consequently, the role of NC-aPLs positivity as an independent risk factor for reduced birth weight remains inconclusive, necessitating further validation through studies with larger sample sizes.

In our study, patients who tested positive for NC-aPLs received significantly more intensive pharmacotherapy, including LMWH, HCQ, and glucocorticoids. Multivariable regression analysis showed that medication intensity was significantly associated with lower odds of APO (1–2 drugs: aOR =  0.177, 95% CI 0.055–0.569, *P* = 0.004; 3–4 drugs: aOR = 0.102, 95% CI 0.037–0.283, *P* < 0.001). This finding aligns with existing literature. For instance, Daniel et al. demonstrated that pregnant patients positive for aPS/PT who were treated with LMWH, aspirin, and/or HCQ successfully achieved live births (28). Similarly, a large European multicenter study involving 187 seronegative APS patients, all of whom carried at least one NC-aPLs, and 285 seropositive APS patients, found that treatment with LDA or a combination of LDA and LMWH significantly improved obstetrical outcomes in the seronegative subgroup (29). Furthermore, Qu et al. provided evidence for the efficacy of a glucocorticoid and LDA combination therapy. In their retrospective analysis of 171 women with unexplained RPL, they found that a regimen combining LDA, prednisone (5 mg/d), and multivitamins resulted in a significantly higher success rate compared to folic acid alone (86.3% vs. 53.3%, *P* < 0.001) (30). Collectively, these findings suggest that our null results may be confounded by the intensive treatment, potentially obscuring the effect of NC-aPLs. Furthermore, stratified analyses based on treatment intensity revealed that an increase in medication intensity was associated with lower odds of overall APO in both NC-aPLs-positive and NC-aPLs-negative groups, thereby providing additional support for this finding.

The reliance on a single-time LIA for antibody detection may have also influenced our findings. Although LIA is extensively utilized for the screening of NC-aPLs and allows for multiplex screening (31), it is characterized by higher sensitivity and lower standardization compared to ELISA (32). This discrepancy may have led to increased false positive rates in both groups, potentially obscuring differences between them. This issue is further exacerbated by our failure to adhere to the 12-week confirmatory testing interval recommended by international APS classification criteria. Transient antibody elevations, which can occur due to infections, inflammation, or medications, might be mistakenly interpreted as true positivity (33). Yonezawa et al. demonstrated that a single positive aPE test lacks predictive value for RPL, whereas persistent positivity upon retesting at 12 weeks is significantly associated with an increased miscarriage rate (34). Despite these limitations, the use of single testing for NC-aPLs remains a straightforward and cost-effective approach, and a positive result can still provide incremental value for diagnosis and risk assessment in routine clinical practice (7, 35). Notably, our single-time-point testing protocol is consistent with that used in most published studies (36). Furthermore, the limited statistical power may explain the null results. A *post hoc* power analysis, conducted using the observed effect size and the final sample size (*α* = 0.05) indicated that the power for detecting the primary APO was below the recommended threshold of 0.80. This insufficient power increases the risk of a type II error, suggesting that a true association between NC-aPLs and APO may have been overlooked in our cohort.

Several limitations of this study warrant consideration. Firstly, the study's single-center, retrospective design may have introduced selection bias. Secondly, the extensive use of pharmacological interventions could have confounded the observed association between NC-aPLs positivity and APO, as such treatments likely mitigated potential risks. Thirdly, the employment of LIA without 12-week confirmatory testing may have elevated false positive rates, thereby obscuring group differences. Furthermore, as aPS/PT IgG and aPA IgM did not produce any positive outcomes, our conclusions concerning aPS/PT and aPA are limited to the analysis of aPS/PT IgM and aPA IgG, respectively. Fourthly, the relatively limited sample size constrained the statistical power necessary to detect subtle effects. Fourthly, the relatively limited sample size constrained the statistical power necessary to detect subtle effects. In addition, the significant result of the Hosmer-Lemeshow test (*P* = 0.019) indicated a potential lack of model fit. Thus, the results should be interpreted with caution. Fifthly, the absence of fetal chromosomal testing left abnormal karyotype, a significant cause of pregnancy loss unaccounted for, potentially confounding the attribution of outcomes. Sixthly, the lack of a healthy control group hindered the assessment of baseline NC-aPLs prevalence in the general population. Future research should prioritize multicenter designs that include standardized therapeutic protocols, confirmatory testing, fetal chromosomal analysis, and healthy comparator groups to minimize confounding bias.

## 5 Discussion

This study demonstrates that NC-aPLs are frequently detected in patients with RPL. After adjusting for confounding variables, no independent association was observed between NC-aPLs and APO. Notably, patients who tested positive for NC-aPLs received more intensive pharmacotherapy, and this treatment intensity was associated with lower odds of APO, which may have masked the potential effect of NC-aPLs. Additionally, the limited sample size reduced the statistical power, and the use of a single-time LIA could not eliminate bias from transient seropositivity, possibly confounding our results. As a result, a potential association cannot be entirely ruled out. Future prospective studies with larger sample sizes, standardized therapeutic protocols, and repeated antibody testing are essential to elucidate the clinical relevance of NC-aPLs and APO in RPL populations.

## Data Availability

The original contributions presented in the study are included in the article/Supplementary Material, further inquiries can be directed to the corresponding authors.
